# Infection status and clinical characteristics of COVID-19 in maintenance hemodialysis patients in Wuhan during the omicron pandemic

**DOI:** 10.1097/MD.0000000000035063

**Published:** 2023-09-08

**Authors:** Hong Liu, Nan Jiang, Yonglong Min, Dan Huang, Huizhen Liu, Hongbo Li, Fei Xiong

**Affiliations:** a Department of Nephrology, Wuhan No. 1 Hospital, Wuhan, China.

**Keywords:** clinical features, COVID-19, epidemic, hemodialysis, mortality

## Abstract

Maintenance hemodialysis (MHD) patients are the high-risk population of infection and death of novel coronavirus disease 2019 (COVID-19), our study aimed to investigate the infection status and clinical characteristics of COVID-19 in MHD patients at a single-center in Wuhan during the Omicron pandemic. In this retrospective, single-center study, we analyzed the clinical data of all MHD patients in Hemodialysis Center of Wuhan No. 1 Hospital from December 2, 2022 to January 6, 2023 during the severe acute respiratory syndrome coronavirus 2 (SARS-CoV-2) Omicron pandemic outbreak. We analyzed the epidemiological and clinical characteristics of deaths and survivors of MHD patients. The infection rate of SARS-CoV-2 in MHD patients was 93.32% (573/614), mortality rate was 8.14% (50/614), hospitalization rate was 23.29% (143/614), and the vaccination rate of COVID-19 was 4.89% (30/614). The median survival time of dead patients was 11 days, the mortality rate of male MHD patients was significantly higher than female. Elderly MHD patients had a higher mortality rate, with a average age of death higher than 70 years old. Additionally, the mortality rate of MHD patients infected with SARS-CoV-2 was higher if the primary disease was hypertensive renal damage or diabetic nephropathy. Laboratory results showed that the lower the albumin level and the higher the C-reactive protein level of MHD patients who died of SARS-CoV-2 infection and severe and critical survival patients. In surviving MHD patients infected with SARS-CoV-2, the most common symptoms were hypodynamia (84.70%), decreased appetite (81.26%) and cough (80.69%). The symptoms of fever, chest tightness and panting, cough, pharyngalgia, hypodynamia, decreased appetite in surviving MHD patients with severe and critical type were significantly higher than those in patients with mild and moderate type. MHD patients are a highly vulnerable population at increased risk of mortality during the Omicron pandemic. Elderly, male, primary disease was hypertensive renal damage or diabetic nephropathy, hypoproteinemia and high C-reactive protein level, all of which will lead to increased mortality in MHD patients.

## 1. Introduction

Coronavirus disease 2019 (COVID-19) is overwhelming healthcare systems worldwide, and despite significant advances in clinical research, the continued spread of severe acute respiratory syndrome coronavirus 2 (SARS-CoV-2) virus and its variants remains a concern.^[1]^ The COVID-19 pandemic represents an unprecedented challenge for patients requiring maintenance hemodialysis (MHD). Numerous studies have shown that MHD patients are at significantly higher risk of COVID-19 infection, serious complications and even death than the general population due to advanced age, more underlying disease, malnutrition, and the need for 2 to 3 weekly routine hemodialysis.^[2]^

From the initial year of the epidemic, 2020, until the end of 2022, China has taken the lead in adopting a zero-tolerance approach to the new coronary pneumonia epidemic, with better outcomes and lower infection rates than most countries in the world.^[3]^ In December 2022, China experienced a major outbreak of neo-crown pneumonia with an unprecedented peak of Omicron infection.^[4]^ The Airfinity model predicts that 1.7 million people are expected to die from COVID-19 in China by the end of April 2023,^[4]^ but studies assessing the infection rate and prognosis of hemodialysis patients at the peak of this outbreak are rarely reported. Therefore, studies are urgently needed to describe the infection status, outcomes, and associated risk factors of Chinese MHD patients after the elimination of normative nucleic acid testing to help in the COVID-19-related medical management of this patient population. Our study analyzed data from 614 MHD patients at a single-center medical institution in Wuhan and found that this group of patients was at greater risk for COVID-19 infection and had higher rates of severe illness and mortality.

## 2. Materials and methods

### 2.1. Study design and participants

Retrospective analysis was made on the clinical data of all MHD patients in Hemodialysis Center of Wuhan No. 1 Hospital from December 2, 2022 to January 6, 2023 during the SARS-CoV-2 Omicron pandemic outbreak. There were 620 cases in total, of which 6 died of non-SARS-CoV-2 infection and were excluded. The clinical data of 614 MHD patients were statistically analyzed. According to the outcome, MHD patients were divided into positive death group, positive survival group and negative survival group. COVID-19 was diagnosed according to the New Coronavirus Pneumonia Prevention and Control Program (9th edition) published by the National Health Commission of China.^[5]^ The study was approved by the Ethics Committees of Wuhan No.1 Hospital. This article is a retrospective study exempting written informed consent but obtained verbal consent obtained.

### 2.2. Data collection

The basic characteristics and laboratory examination data of MHD patients were obtained from the system of Wuhan Hemodialysis Quality Control Center. Basic characteristic data including sex, age, dialysis age, hemodialysis access, and primary disease, laboratory examination data including hemoglobin (Hb), albumin (Alb), calcium, phosphorus, parathyroid hormone and C-reactive protein (CRP); the clinical manifestation data of positive and negative survival patients were obtained by hemodialysis doctor after investigating each patient, including fever, chest tightness and panting, cough, pharyngalgia, hypodynamia, myodynia and arthrodynia, decreased appetite, diarrhea, anosmia, and ageusia, the clinical manifestation data endpoint was monitored up to January 6, 2023. All data were cross-checked by 2 hemodialysis doctor.

### 2.3. Definitions

After nucleic acid testing (NAT), with positive NAT were considered to be infected with COVID-19 and negative NAT were considered to be non-COVID-19 infected. Positive death refers to MHD patients who died after infected with COVID-19, Positive survival refers to survival MHD patients after infected with COVID-19, Negative survival refers to survival MHD patients who have not infected with COVID-19. Fever was defined as a temperature of at least 37.3°C. Fever grade: low-grade fever: 37.3 to 38°C; moderate fever: 38.1 to 39°C; high-grade fever: 39.1 to 41°C; Ultra high fever: above 41°C.

### 2.4. Clinical classification

The degree of severity of COVID-19 (mild vs moderate vs severe vs critical) was defined according to the New Coronavirus Pneumonia Prevention and Control Program (9th edition).^[5]^ Mild type: Mild clinical symptoms, no viral pneumonia manifestations on chest CT scans; Moderate type: with fever, respiratory tract and other symptoms, and pneumonia manifestations on chest CT scans; Severe type: shortness of breath, respiratory rate > 30 breaths/minutes; oxygen saturation < 93% at rest state; arterial blood oxygen partial pressure (PaO_2_)/oxygen concentration (FiO_2_) ≤ 300 mm Hg (1 mm Hg = 0.133 kPa). At high altitude (over 1000 meters above sea level), PaO_2_/FiO_2_ should be corrected according to the following formula: PaO_2_/FiO_2_ × [atmospheric pressure (mm Hg)/760]. Pulmonary lesion progression > 50% within 24 to 48 hours on radiologic imaging were treated as severe cases; Critical type: Those who meet any of the following criteria: Occurrence of respiratory failure requiring mechanical ventilation; Presence of shock; Other organ failure that requires monitoring and treatment in the intensive care unit.

### 2.5. Statistical analysis

SPSS24.0 software (SPSS Inc., Chicago, IL) was used for statistical description and analysis of the data. The measurement data was expressed by $\bar{x}\pm s$, and groups comparison were performed by 1-way ANOVA analysis or Kruskal–Wallis test. Count data was expressed in frequency (%), and Pearson chi-square test was used for comparison between groups. Survival curve analysis was presented by the Kaplan–Meier method, and the Log-rank test verifed any diference. *P* < .05 was considered statistically significant.

## 3. Results

### 3.1. Basic characteristics of MHD patients

From all the 620 MHD patients in Hemodialysis Center of Wuhan No. 1 Hospital, of which 6 died of non-SARS-CoV-2 infection and were excluded. The clinical data of 614 MHD patients were statistically analyzed. The infection rate of SARS-CoV-2 in MHD patients was 93.32% (573/614), among which 50 (8.14%) positive death patients. Three hundred fifty-eight (58.31%) were male and 256 (41.69%) were female, the positive mortality rate of male versus female was 10.34% versus 5.08%% (*P* < .05), suggesting that the higher positive mortality of male patients. The average age of MHD patients was 60.73 ± 12.78 years, of which the positive death patients was 71.90 ± 10.37 years, compared with positive and negative survival patients (*P* < .001), suggesting that the older the positive death patients. In the age distribution, 356 (57.98%) were younger than 65 years had a positive mortality rate of 2.53%, 258 (42.02%) aged 65 years or older had a positive mortality rate of 15.89% (*P* < .001), suggesting that the older the age, the higher the mortality rate after infection; however, there is no significant statistical difference between the 3 groups in terms of average dialysis age and hemodialysis access. In terms of primary disease of MHD patients, 326 (53.09%) with primary glomerular disease, including 19 (5.83%) positive death patients, 103 (16.78%) with hypertensive renal damage, including 16 (15.53%) positive death patients, 110 (17.92%) with diabetic nephropathy, including 12 (10.91%) positive death patients, and 75 (12.21%) with other primary diseases, including 3 (4.00%) positive death patients (*P* < .05), suggesting that the positive mortality of patients infected with SARS-CoV-2 was higher if the primary disease was hypertensive renal damage or diabetic nephropathy (Table 1).

**Table 1:** Basic characteristics of MHD patients. Values expressed by $\bar{x}\pm s$ or n (%).

		Total (n = 614)	Positive death (n = 50)	Positive survival (n = 523)	Negative survival (n = 41)	Statistical value	*P* value
Sex	Male	358 (58.31)	37 (10.34)	301 (84.08)	20 (5.58)	6.72	.035*
	Female	256 (41.69)	13 (5.08)	222 (86.72)	21 (8.20)		
Age (yr)		60.73 ± 12.78	71.90 ± 10.37	59.70 ± 12.40	61.24 ± 14.04	22.27	<.001†
	x < 65	356 (57.98)	9 (2.53)	321 (90.17)	26 (7.30)	35.78	<.001†
	x ≥ 65	258 (42.02)	41 (15.89)	202 (78.29)	15 (5.81)		
Dialysis age (yr)		6.06 ± 4.88	6.21 ± 4.57	6.09 ± 4.90	5.43 ± 5.11	1.97	.372
	x ≤ 5	306 (49.84)	24 (7.84)	260 (84.97)	22 (7.19)	1.80	.773
	5 < x ≤ 10	205 (33.39)	20 (9.76)	172 (83.90)	13 (6.34)		
	x > 10	103 (16.77)	6 (5.83)	91 (88.35)	6 (5.83)		
Hemodialysis access	Internal fistula	489 (79.64)	35 (7.16)	421 (86.09)	33 (6.75)	3.47	.483
	Long-term catheter	116 (18.89)	14 (12.07)	95 (81.90)	7 (6.03)		
	Temporary catheter	9 (1.47)	1 (11.11)	7 (77.78)	1 (11.11)		
Primary disease	Primary glomerular disease	326 (53.09)	19 (5.83)	287 (88.04)	20 (6.13)	16.15	.013*
	Hypertensive renal damage	103 (16.78)	16 (15.53)	83 (80.58)	4 (3.88)		
	Diabetic nephropathy	110 (17.92)	12 (10.91)	87 (79.09)	11 (10.00)		
	Others	75 (12.21)	3 (4.00)	66 (88.00)	6 (8.00)		

MHD = Maintenance hemodialysis.

* *P* < .05.

† *P* < .01.

### 3.2. Laboratory examination of MHD patients

The laboratory examination results showed that the average Alb of MHD patients was 38.05 ± 4.89 g/L, of which the positive death patients was 36.17 ± 5.25 g/L, significantly lower than the other 2 groups (*P* < .05), suggesting that the lower the albumin level of MHD patients who died of SARS-CoV-2 infection; 240 (39.09%) with Alb ≥ 40 g/L had a positive mortality rate of 4.58%, and 374 (60.91%) with Alb < 40 g/L had a positive mortality rate of 10.43% (*P* < .05), suggesting that the lower the albumin level, the higher the positive mortality rate. The average CRP of MHD patients was 20.40 ± 40.19 mg/L, of which the positive death patients was 49.29 ± 78.80 mg/L, significantly higher than the other 2 groups (*P* < .001), suggesting that the higher the CRP level of MHD patients who died of SARS-CoV-2 infection. Two hundred seventy-nine (45.44%) with normal CRP had a positive mortality rate of 4.30%, 335 (54.56%) with elevated CRP had a positive mortality rate of 11.34% (*P* < .01), suggesting that the higher the level of CRP, the higher the positive mortality rate. However, there was no significant difference in the results of Hb, calcium, phosphorus and parathyroid hormone (*P* > .05), suggesting that there was no significant difference in these indicators whether MHD patients were infected with SARS-CoV-2 (Table 2).

**Table 2:** Laboratory examination of MHD patients. Values expressed by $\bar{x}\pm s$ or n (%).

		Total (n = 614)	Positive death (n = 50)	Positive survival (n = 523)	Negative survival (n = 41)	Statistical value	*P* value
Hb (g/L)		106.93 ± 18.96	109.80 ± 23.72	106.41 ± 18.42	109.98 ± 19.25	3.39	.184
	Hb ≥ 110	287 (46.74)	28 (9.76)	235 (81.88)	24 (8.36)	4.70	.095
	Hb < 110	327 (53.26)	22 (6.72)	288 (88.07)	17 (5.20)		
Alb (g/L)		38.05 ± 4.89	36.17 ± 5.25	38.23 ± 4.85	38.14 ± 4.57	8.40	.015*
	Alb ≥ 40	240 (39.09)	11 (4.58)	214 (89.17)	15 (6.25)	6.98	.031*
	Alb < 40	374 (60.91)	39 (10.43)	309 (82.62)	26 (6.95)		
Ca (mmol/L)		2.21 ± 0.23	2.17 ± 0.21	2.22 ± 0.23	2.19 ± 0.25	1.25	.287
	2.1 ≤ Ca ≤ 2.5	357 (58.14)	29 (8.12)	301 (84.31)	27 (7.56)	1.08	.584
	Ca < 2.1 or Ca > 2.5	257 (41.86)	21 (8.17)	222 (86.38)	14 (5.45)		
P (mmol/L)		1.58 ± 0.55	1.54 ± 0.70	1.57 ± 0.51	1.65 ± 0.68	1.96	.376
	*P* ≤ 1.78	417 (67.92)	40 (9.59)	350 (83.93)	27 (6.47)	3.67	.160
	*P* > 1.78	197 (32.08)	10 (5.08)	173 (87.82)	14 (7.11)		
PTH (ng/L)		372.98 ± 392.45	319.94 ± 440.95	374.48 ± 389.71	418.46 ± 366.81	5.79	.055
	PTH ≤ 600	502 (81.76)	45 (8.96)	428 (85.26)	29 (5.78)	5.62	.060
	PTH > 600	112 (18.24)	5 (4.46)	95 (84.82)	12 (10.71)		
CRP (mg/L)		20.40 ± 40.19	49.29 ± 78.80	18.22 ± 34.68	13.08 ± 17.13	14.38	<.001†
	Normal CRP	279 (45.44)	12 (4.30)	247 (88.53)	20 (7.17)	10.13	.006†
	Elevated CRP	335 (54.56)	38 (11.34)	276 (82.39)	21 (6.27)		

Alb = albumin, Ca = calcium, CRP = C-reactive protein, Hb = hemoglobin, MHD = Maintenance hemodialysis, P = phosphorus, PTH = parathyroid hormone.

* *P* < .05.

† *P* < .01.

### 3.3. Survival curve for positive death patients

**Figure 1.**
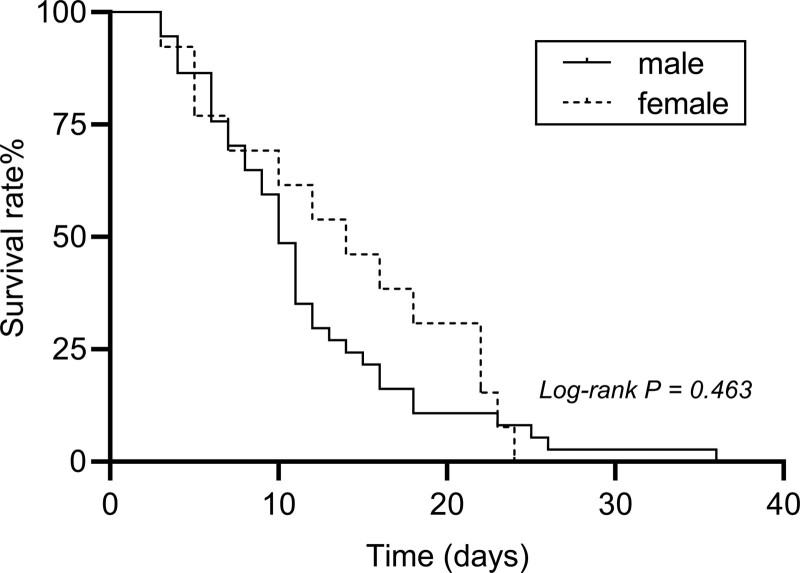
Survival curve of positive death patients.

After SARS-CoV-2 infection in MHD patients, the median survival time to 50 positive death patients was 11 days (95% confidence interval [CI] 9.63–12.36), of which median survival time to 37 male positive death patients was 10 days (95% CI 8.68–11.32), 13 female positive death patients was 14 days (95% CI 6.95–21.05), there was no significant statistical difference (Log-rank *P* = .463 > 0.05) (Fig. 1).

### 3.4. Basic characteristics and clinical manifestation of positive survival patients

The incidence of severe and critical type in MHD patients younger than 65 years versus 65 years or older was 16.51% versus 35.15% (*P* < .001), suggesting that the older the age, the higher the incidence of severe and critical type, however, there was no significant difference in sex, dialysis age, hemodialysis access and primary disease (*P* > .05). The symptoms of fever, chest tightness and panting, cough, pharyngalgia, hypodynamia, decreased appetite in MHD patients with severe and critical type were significantly higher than those in patients with mild and moderate type (*P* < .01), but there was no statistical difference in the clinical manifestations of myodynia and arthrodynia, diarrhea, anosmia and ageusia (*P* > .05). The incidence of severe and critical type in MHD patients without anemia versus with anemia was 17.45% versus 28.82% (*P* = .002), the incidence of severe and critical type in MHD patients without Alb decreased versus with Alb decreased was 14.49% versus 30.10% (*P* < .001), the incidence of severe and critical type in MHD patients without elevated CRP versus with elevated CRP was 12.96% versus 33.33% (*P* < .001), all of which mean that the more severe anemia, the lower albumin, and the more severe inflammatory reaction in severe and critical type patients with SARS-CoV-2 infection (Table 3).

**Table 3:** Basic characteristics and clinical manifestation of positive survival patients. Values expressed by n (%).

		Total (n = 523)	Mild and moderate type (n = 399)	Severe and critical type (n = 124)	Severe and critical type(%)	*X^2^*	*P* value
Sex	Male	301	230	71	23.59%	0.01	.940
	Female	222	169	53	23.87%		
Age (yr)	X < 65	321	268	53	16.51%	23.80	<.001*
	X ≥ 65	202	131	71	35.15%		
Dialysis age (yr)	X ≤ 5	260	193	67	25.77%	1.36	.505
	5 < x ≤ 10	172	136	36	20.93%		
	X > 10	91	70	21	23.08%		
Hemodialysis access	Internal fistula	421	329	92	21.85%	4.66	.097
	Long-term catheter	95	66	29	30.53%		
	Temporary catheter	7	4	3	42.86%		
Primary disease	Primary glomerular disease	288	230	58	20.14%	7.74	.052
	Hypertensive renal damage	83	62	21	25.30%		
	Diabetic nephropathy	87	57	30	34.48%		
	Others	65	50	15	23.08%		
Fever	No	280	235	45	16.07%	19.44	<.001*
	Yes	243	164	79	32.51%		
Chest tightness and panting	No	304	300	4	1.32%	201.27	<.001*
	Yes	219	99	120	54.80%		
Cough	No	101	92	9	8.91%	15.15	<.001*
	Yes	422	307	115	27.25%		
Pharyngalgia	No	315	259	56	17.78%	15.41	<.001*
	Yes	208	140	68	32.69%		
Hypodynamia	No	80	80	0	0.00%	29.35	<.001*
	Yes	443	319	124	27.99%		
Myodynia and arthrodynia	No	323	254	69	21.36%	2.57	.109
	Yes	200	145	55	27.50%		
Decreased appetite	No	98	85	13	13.27%	7.27	.007*
	Yes	425	314	111	26.12%		
Diarrhoea	No	403	315	88	21.84%	3.41	.065
	Yes	120	84	36	30.00%		
Anosmia	No	437	328	109	24.94%	2.24	.135
	Yes	86	71	15	17.44%		
Ageusia	No	405	304	101	24.94%	1.50	.221
	Yes	118	95	23	19.49%		
Anemia	No	235	194	41	17.45%	9.25	.002*
	Yes	288	205	83	28.82%		
Decreased Alb	No	214	183	31	14.49%	17.04	<.001*
	Yes	309	216	93	30.10%		
Elevated CRP	No	247	215	32	12.96%	29.92	<.001*
	Yes	276	184	92	33.33%		

Alb = albumin, CRP = C-reactive protein.

* *P* < .01.

## 4. Discussion

On December 2, 2022, the first case of SARS-CoV-2 infection occurred in our dialysis center. On December 7, 2022, China adjusted public health control measures, SARS-CoV-2 infection was widespread in Chinese Mainland.^[6]^ In December 2022, Hubei Provincial Center for Disease Control and Prevention carried out the surveillance of Omicron variant on outpatient and inpatient cases in medical institutions. The results showed that the genotype of COVID-19 virus in the whole province was BA.5.2 and BF.7, of which BA.5.2 accounted for 89% and BF.7 accounted for 11%. In a short time, a large number of hemodialysis patients infected with Omicron appeared in our dialysis center, the peak of Omicron infection did not end until January 6, 2023. This research presented and analyzed the infection status and death, clinical characteristics of COVID-19 in all MHD patients in our dialysis center during the Omicron pandemic.

MHD patients may present a higher incidence and higher risk of death due to their low immunity than the general population after infection with SARS-CoV-2.^[7–9]^ A meta-analysis of more than 396,000 patients on MHD patients reported that a SARS-CoV-2 incidence of 7.7% (95% CI 5.0–10.9%) and a mortality rate of 22.4% (95% CI 17.9–27.1%).^[8]^ Our previous cohort study showed that the mortality rate of MHD patients infected with the original SARS-CoV-2 strain was 39.2%,^[10]^ which was much higher than that of the general population in the same city (8%).^[11]^ For the current epidemic Omicron variant, our study observed that the incidence of infection in MHD patients was 93.32% (573/614), and the mortality rate was 8.14% (50/614). The incidence rate was significantly higher than that of the original SARS-CoV-2 strain, but the mortality rate was markedly decline, which was related to the increase of the immune escape ability of the virus and the significant reduction of the mortality rate caused by the virus mutation. Noteworthy, although the incidence of Omicron virus infection in MHD patients was not significantly different from that in the general population, but the mortality rate was significantly higher. We analyzed the causes of death and complications of 50 patients who died of COVID-19 infection. Among them, 4 cases (8%) died suddenly after COVID-19 infection, 15 cases (30%) died of COVID-19 severe pneumonia, 26 cases (52%) of COVID-19 complicated with acute cardiovascular disease, 3 cases (6%) of COVID-19 complicated with acute cerebral infarction, and 2 cases (4%) of COVID-19 complicated with gastrointestinal bleeding. It can be seen that cardiovascular and cerebrovascular diseases after COVID-19 infection are very common and need attention. Additionally, studies have reported that 28.6% to 82.5% of MHD patients infected with SARS-CoV-2 required hospitalization and about 6.6% to 28.6% intensive care.^[8,12–14]^ In our study, about 23.29% (143/614) of MHD patients were hospitalized after infection with Omicron, which was significantly higher than the general population. This study also showed that the median survival time of 50 dead patients was 11 days, while male (10 days) was shorter than female (14 days), which indicated that SARS-CoV-2 infection may lead to rapid disease progress and death in a relatively short time that needed urgent attention.

Consistent with previous reports,^[15,16]^ our data showed that the mortality rate of male MHD patients was significantly higher than female after infection with SARS-CoV-2, we speculated that it may be related to worse fluid volume management and stronger tolerance in males, which leading to more neglect the importance of COVID-19 in male MHD patients. Worldwide, the risk of death of the elderly infected with SARS-CoV-2 is significantly higher.^[17]^ Similarly, in the hemodialysis population, the mortality rate of the elderly was higher, with a average age of death higher than 70 years old. Among the surviving patients the higher the incidence rate of severe and critical type in the elderly, which may be related to the more severe underlying diseases and lower immunity of elderly patients. Additionally, the positive mortality of MHD patients infected with SARS-CoV-2 was higher if the primary disease was hypertensive renal damage or diabetic nephropathy, which may be related to hypertension and diabetes are the most common risk factors for cardiovascular disease. It is noteworthy that although there was no statistical significance in terms of dialysis age and hemodialysis access, however, the death percentage was significantly higher in catheter group. We speculated that it may be related to the worse cardiovascular conditions of MHD patients who use catheters, as well as the micro-inflammatory, larger sample studies may be needed to confirm.

In hemodialysis patients, hypo hemoglobinemia and hypoalbuminemia are risk factors for death.^[18]^ Although we did not find that positive death patients had lower Hb than surviving patients, the probability of conversion to severe and critical illness was significantly higher in positive surviving patients with anemia than those without anemia, implying that anemia is a risk factor for disease exacerbation. In terms of albumin levels, positive death patients had significantly lower albumin levels than surviving patients, meanwhile, the probability of conversion to severe and critical illness was significantly higher in positive surviving patients with low albumin level than those with normal albumin level. These findings indicated that patients with poor nutrition have lower immunity, worse prognosis, and higher mortality rate. As a result, we should pay more attention to nutritional index monitoring and management in hemodialysis patients. Inflammatory factor storm has been clearly demonstrated in patients with SARS-CoV-2 infection, consistent with previous reports,^[19]^ our results also showed that CRP levels were higher in MHD patients who died of SARS-CoV-2 infection. Among surviving patients, the probability of conversion to severe and critical illness was significantly higher with elevated CRP than those with normal CRP, suggesting a more severely inflammatory response in severe and critical type patients. Therefore, more emphasis should be placed on antiinfective and anti-inflammatory during treatment.

We also investigated the clinical manifestations of positive survival patients, the most common symptoms were hypodynamia (84.70%), decreased appetite (81.26%) and cough (80.69%). Due to the rapid outbreak of the COVID-19 Omicron and limited medical resources, resulting in a low number of hemodialysis patients having outpatient lung CT, so we were unable to use the severity of lung CT for clinical classification. Finally, we performed a statistical comparison using oxygen saturation below 93% as the mild and moderate type, oxygen saturation above 93% as the severe and critical type. Our results showed that the symptoms of fever, chest tightness and panting, cough, pharyngalgia, hypodynamia, decreased appetite in MHD patients with severe and critical type were significantly higher than those in patients with mild and moderate type. Patients with fever accounted for only 46.46% (243/523), of which low-grade fever 19.31% (101/523), moderate fever 20.08% (105/523) and high-grade fever 7.07% (37/523). The median fever time of MHD patients in both groups was 2 days, which was not significantly different from the general population. It is noteworthy that, the majority of MHD patients appeared to show inconspicuous clinical manifestations in early stages, but the oxygen saturation had significantly decreased, which may be related to MHD patients exhibiting a more serious immunosuppression state. This implied that we should pay particular attention to the oxygen saturation of MHD patients, which is conducive to timely and early treatment.

MHD patients are not only more likely to be exposed to SARS-CoV-2 infection, but also have a higher risk of developing severe symptoms and death than the general population. Research has shown that vaccination of MHD patients reduces mortality significantly.^[20]^ The COVID-19 vaccination rate among all MHD patients in our study was 4.89% (30/614), none of the 50 patients who died having received the vaccine. The low vaccination rate and high mortality rate in MHD patients is clearly opposite to that in general population, and the high mortality rate may be related to not only the underlying disease, but also the absence of vaccination, more data are needed for validation.

This study has limitations because it was based on a retrospective single-center design. Additionally, detailed imaging data were not available in all cases, preventing us from performing a more meaningful clinical classification. At the same time, this study lacks the description of treatment measures. In the follow-up, we will analyze the treatment measures, evolution and prognosis, as well as influencing factors of hemodialysis patients infected with COVID-19 to supplement this study.

## 5. Conclusion

The present study indicates that MHD patients remains a highly vulnerable population at increased risk of mortality during the Omicron pandemic. Elderly, male, primary disease was hypertensive renal damage or diabetic nephropathy, hypoproteinemia and high CRP level, all of which will lead to increased mortality in MHD patients. Additionally, the monitoring of oxygen saturation is particularly important for the timely and early treatment of MHD patients. Meanwhile, the most common symptoms such as hypodynamia, decreased appetite and cough should be paid particular attention. These findings provide evidences for better administration and treatment strategies of MHD patients infected with COVID-19.

## Author contributions

**Conceptualization:** Fei Xiong.

**Data curation:** Hong Liu, Nan Jiang, Yonglong Min.

**Formal analysis:** Hong Liu, Yonglong Min.

**Investigation:** Hong Liu, Nan Jiang, Dan Huang, Huizhen Liu, Hongbo Li.

**Methodology:** Hong Liu, Yonglong Min, Fei Xiong.

**Supervision:** Hongbo Li, Fei Xiong.

**Visualization:** Hongbo Li.

**Writing – original draft:** Hong Liu, Nan Jiang.

**Writing – review & editing:** Fei Xiong.
